# Supplementary material for: An unbiased approach to measure aberrant DNA methylation alterations
Source: Nat Commun. 2026 Mar 27;17:4522. doi: 10.1038/s41467-026-71089-5 (PMC13194872; doi:10.1038/s41467-026-71089-5)
Supplement: Supplementary file 1 — Supplementary Information [file 41467_2026_71089_MOESM1_ESM.pdf]

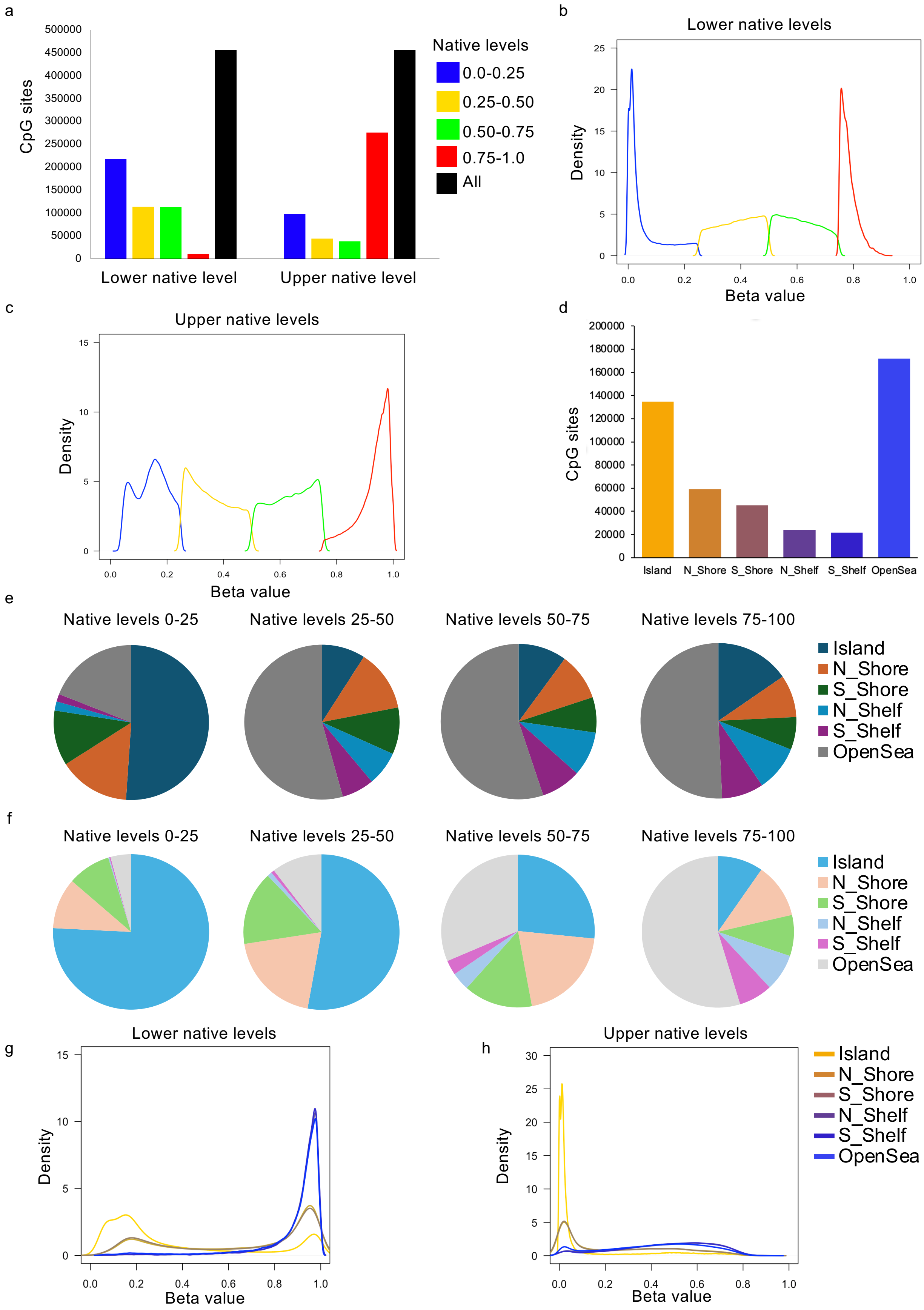

Supplemental Figure 1. Characteristics of native levels in normal tissues. a Bar plot showing the number of CpG sites with lower and upper native DNA methylation levels separated into four quantile intervals. Density plots showing the distribution of the native DNA methylation levels in the **b** lower and **c** upper direction. **d** Bar plot showing the number of CpG sites for the different genomic regions. Pie charts showing the proportion of different genomic regions in the **e** lower and **f** upper direction of the CpG sites with varying quantile intervals of native DNA methylation. Density plots showing the native DNA methylation levels within the different genomic regions in the **g** lower and **h** upper direction.

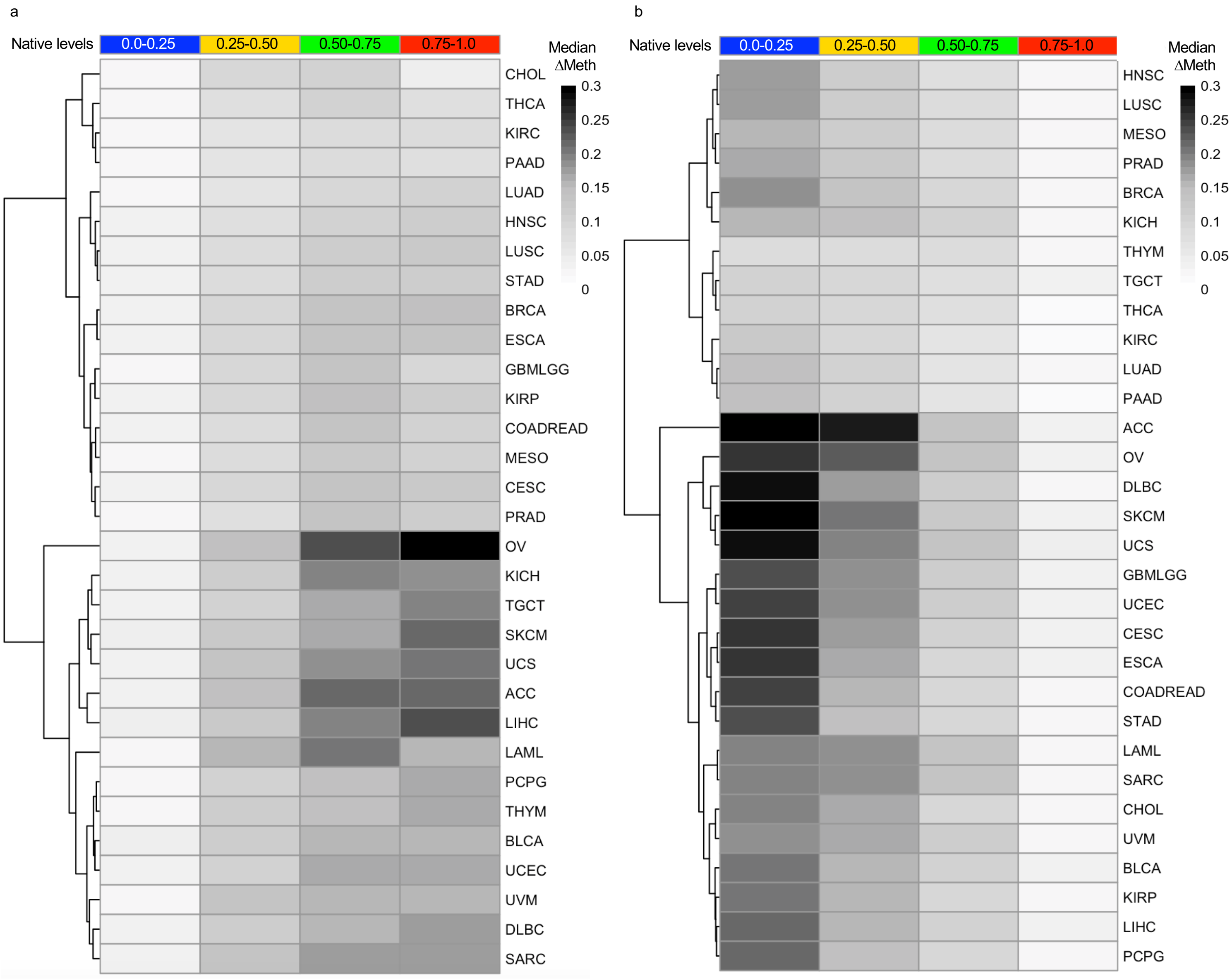

Supplemental Figure 2. Median ΔMeth profiles in tumor tissues. Heatmap of the median ΔMeth of the CpG sites with varying levels of native DNA methylation in the **a** hypomethylation and **b** hypermethylation directions for the 31 tumor types.

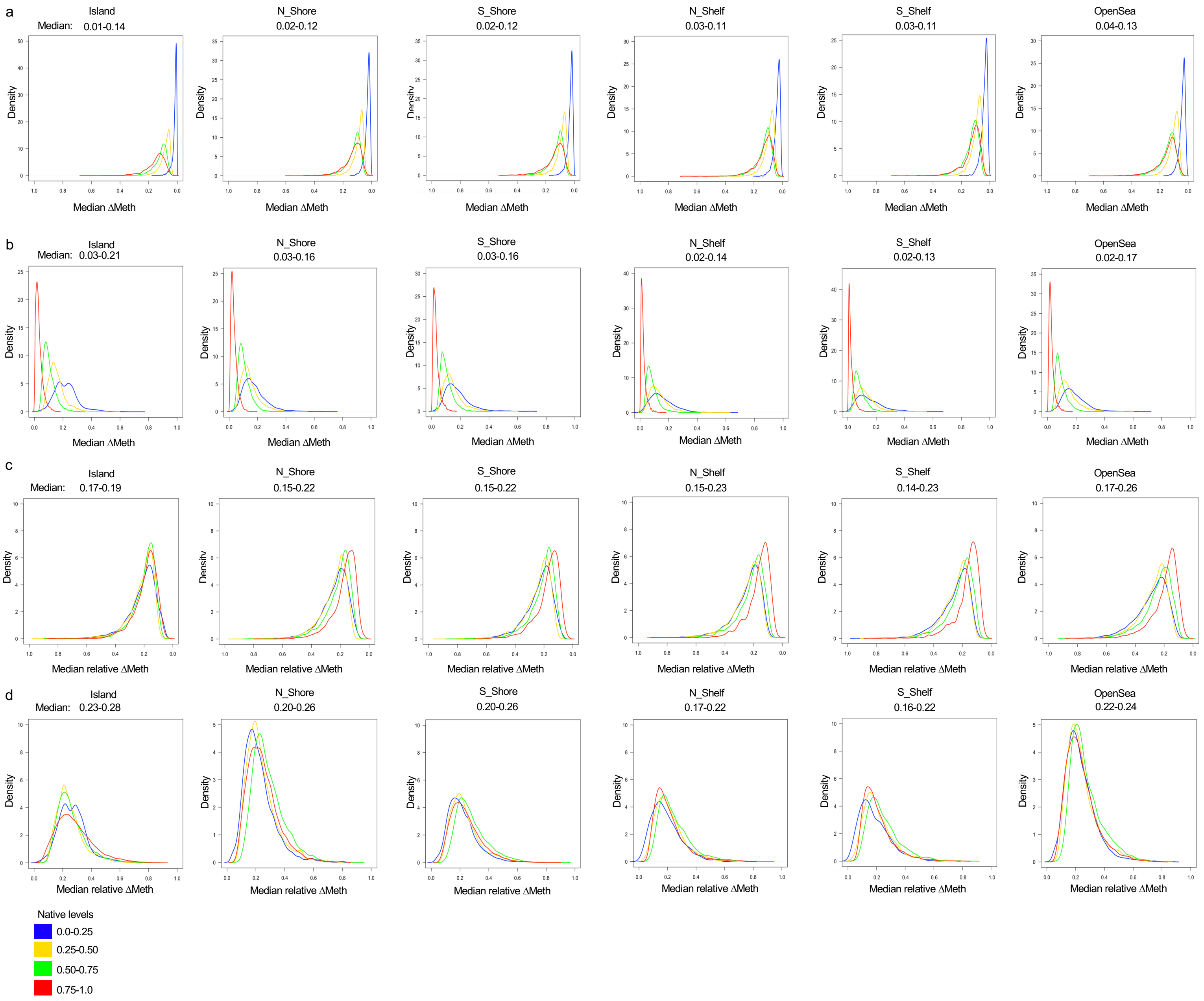

Supplemental Figure 3. Distribution profiles of ΔMeth and relative ΔMeth in tumor tissues at different genomic regions. Distribution of the median ΔMeth in the **a** hypomethylation and **b** hypermethylation direction at different genomic regions. Distribution of the median relative ΔMeth in the **c** hypomethylation and **d** hypermethylation direction at different genomic regions.

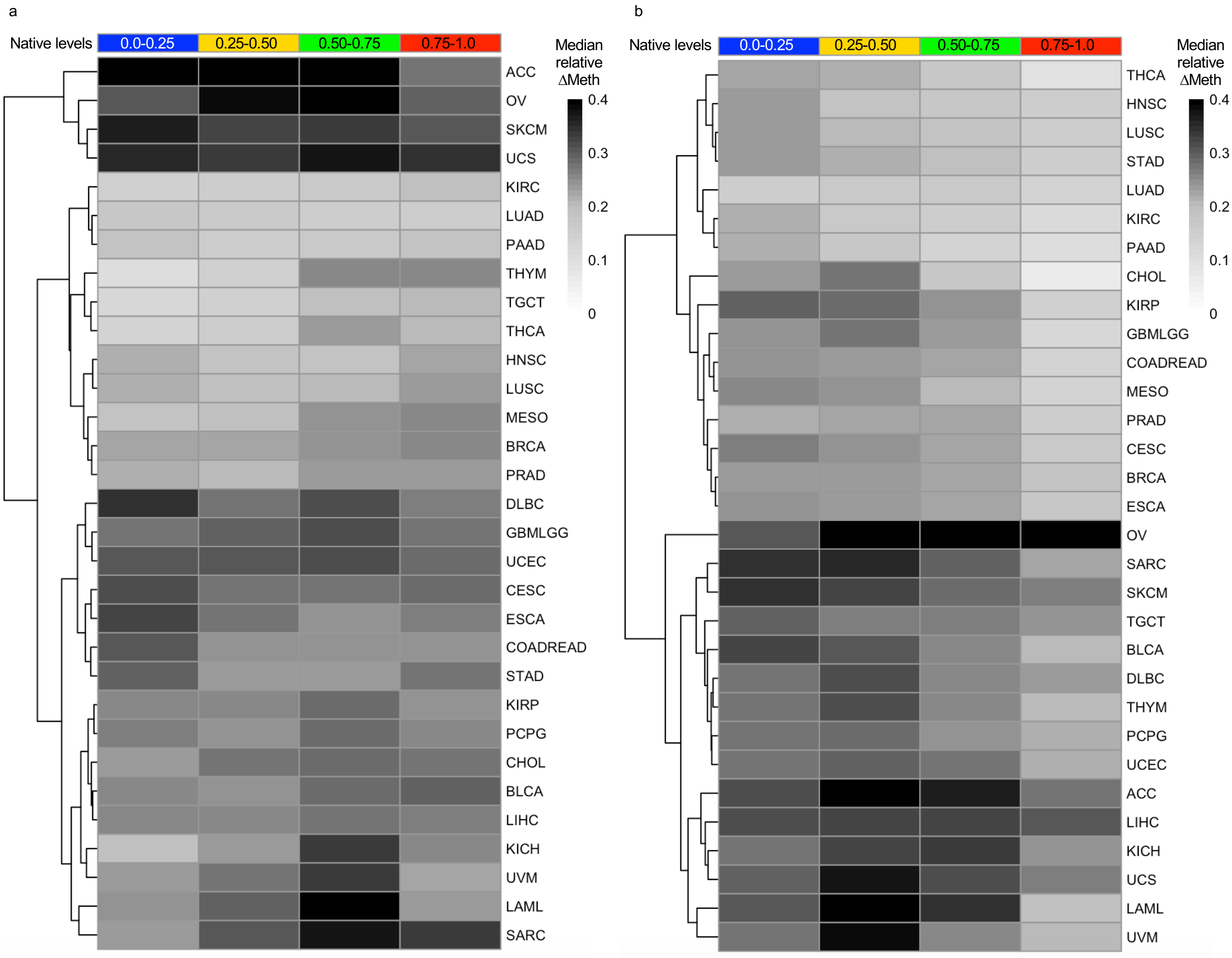

Supplemental Figure 4. Median relative ΔMeth profiles in tumor tissues. Heatmap of the median relative ΔMeth of the CpG sites with varying levels of native DNA methylation in the **a** hypomethylation and **b** hypermethylation directions for the 31 tumor types.

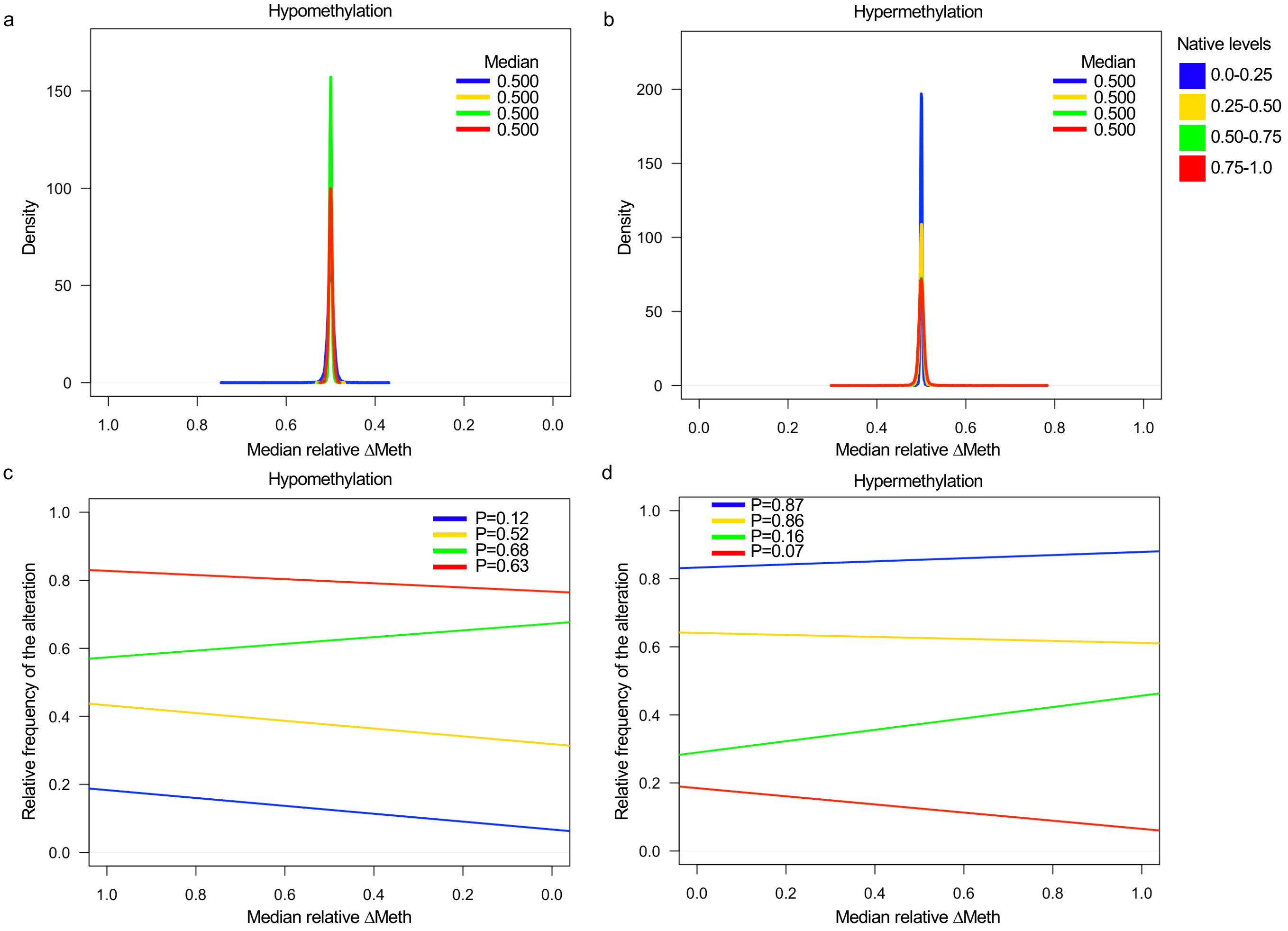

Supplemental Figure 5. Relative ΔMeth profiles in randomly generated DNA methylation dataset. Density plots of the median relative ΔMeth from the randomly generated DNA methylation dataset (n=8,551 samples) in the **a** hypomethylation and **b** hypermethylation directions. Line plots showing the correlation between the proportion of cancer samples with the DNA methylation alteration and the median level of relative ΔMeth from the randomly generated data in the **c** hypomethylation and **d** hypermethylation directions.

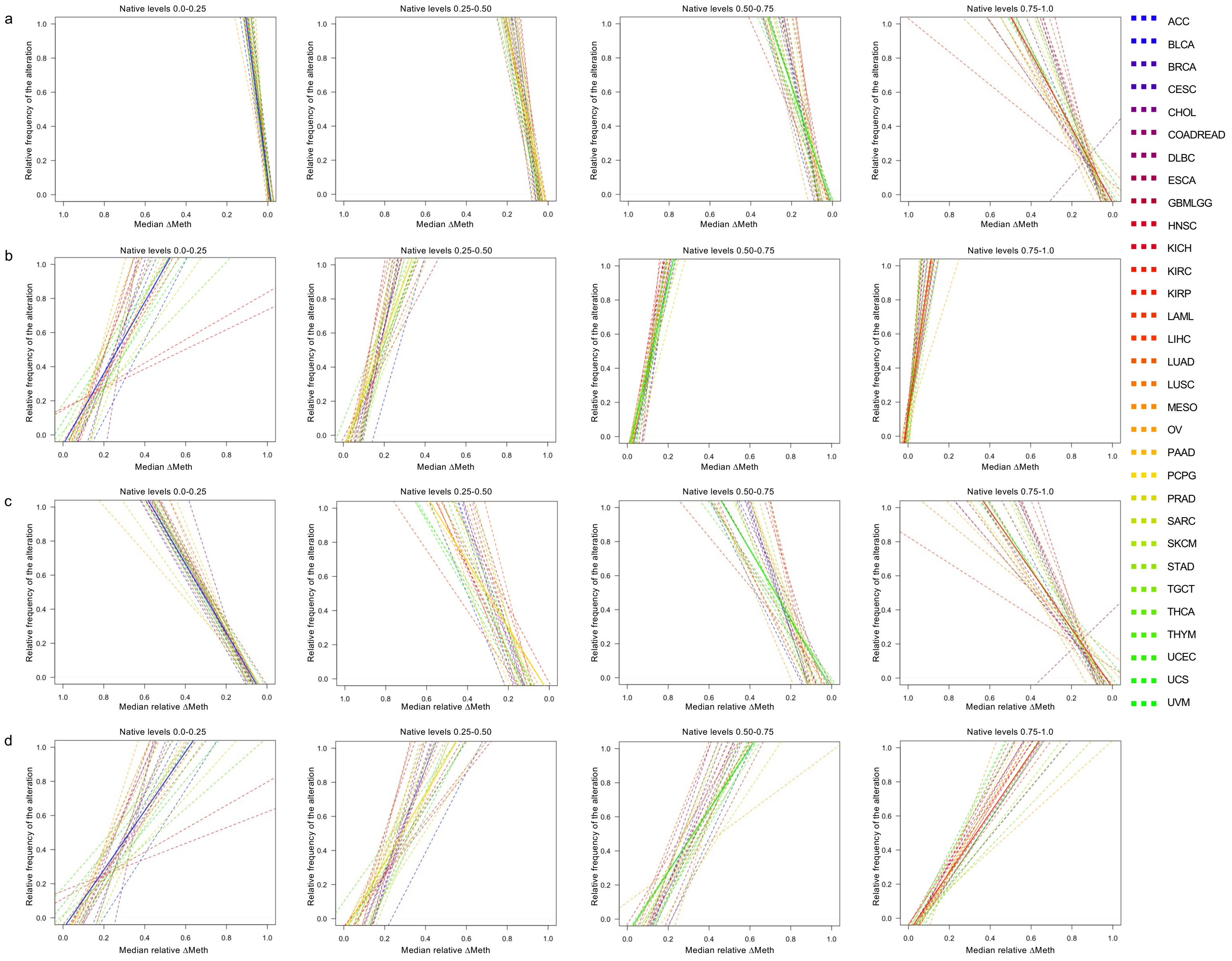

Supplemental Figure 6. Rate of change between the relative frequency of the DNA methylation alteration and the level of the alteration. Line plots showing the correlation between the proportion of cancer samples with the DNA methylation alteration and the level of median ΔMeth in the **a** hypomethylation and **b** hypermethylation directions and the median relative ΔMeth in the **c** hypomethylation and **d** hypermethylation directions. Solid lines represent the median slope.

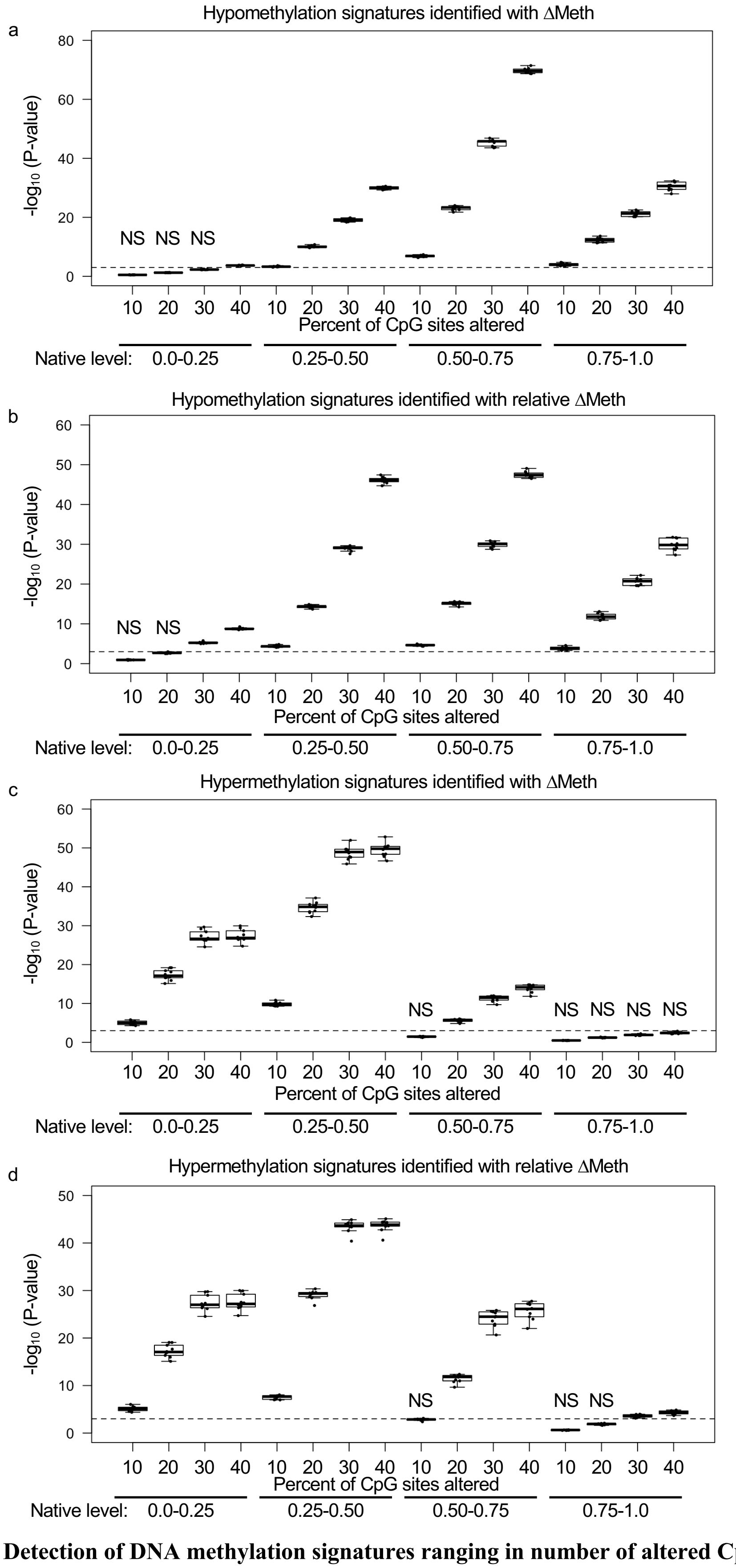

Supplemental Figure 7. Detection of DNA methylation signatures ranging in number of altered CpG sites. Box plots showing the ability of ΔMeth and relative ΔMeth to detect DNA methylation alteration signatures from a dataset of 1000 randomly selected cancer samples. To generate the signatures, we randomly selected a fraction of CpG sites, ranging between 10-40%, and altered them by a relative rate of change of 10% in the hypomethylation direction and measured the ability of **a** ΔMeth and **b** relative ΔMeth to detect the signatures. We also generated hypermethylation signatures and measured ability of **c** ΔMeth and **d** relative ΔMeth to detect the signatures. n=10 for each signature. The P-value (Wilcox rank sum test) was calculated by comparing the generated signature to the native levels. Dash line at P=0.001. NS is not significant P>0.001. Center line, median; box limits, upper and lower quartiles; whiskers, 1.5x interquartile range; points, data.

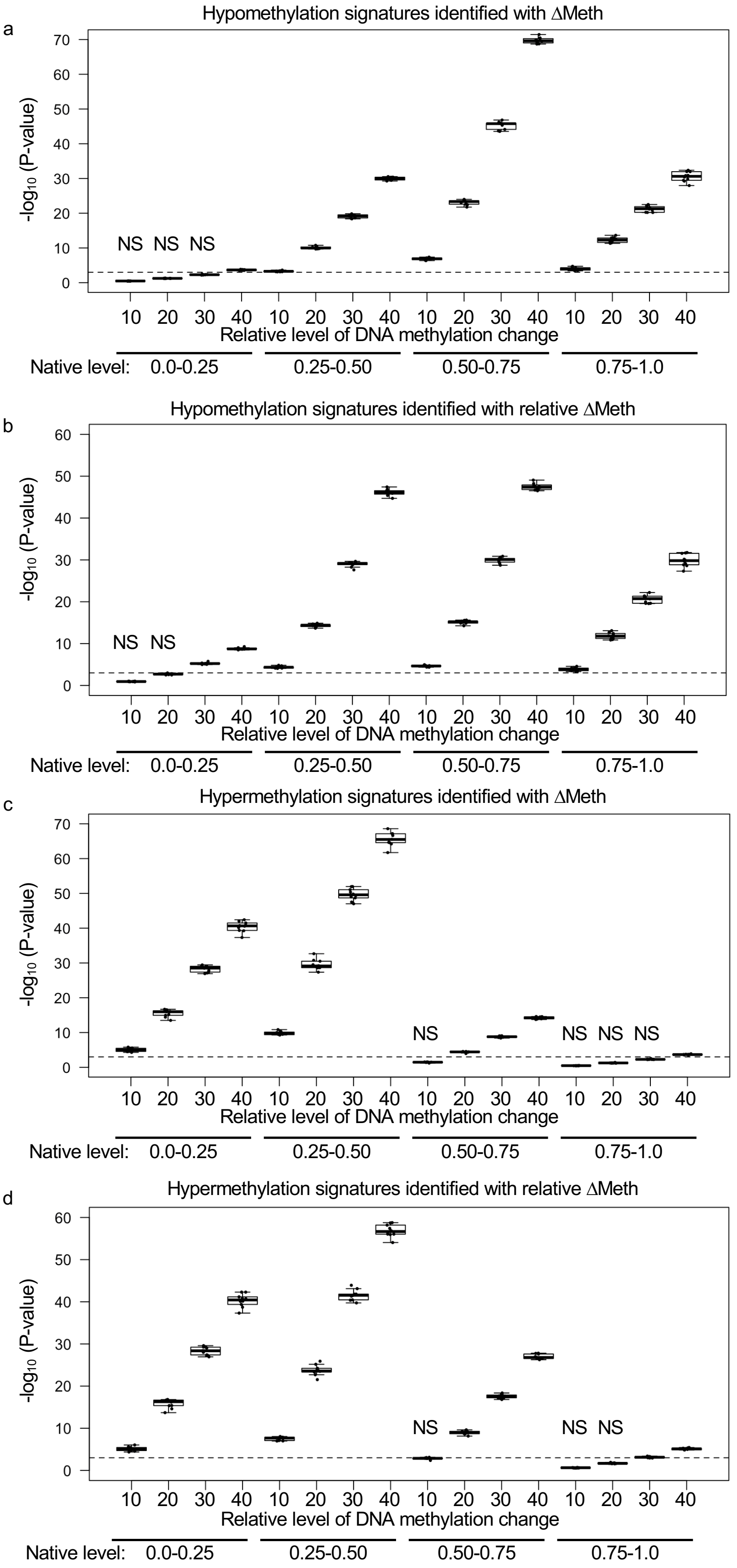

Supplemental Figure 8. Detection of DNA methylation signatures ranging in level of the alteration. Box plots show the ability of ΔMeth and relative ΔMeth to detect DNA methylation alteration signatures from a dataset of 1000 randomly selected cancer samples. To generate the DNA methylation signatures, we randomly selected 10% of the CpG sites and altered them at a relative rate of change ranging between 10-40% in the hypomethylation direction and measured the ability of **a** ΔMeth and **b** relative ΔMeth to detect the DNA methylation signatures. We also generated hypermethylation signatures and measured ability of **c** ΔMeth and **d** relative ΔMeth to detect the signatures. n=10 for each signature. The P-value (Wilcox rank sum test) was calculated by comparing the generated signature to the native levels. Dash line at P=0.001. NS is not significant P>0.001. Center line, median; box limits, upper and lower quartiles; whiskers, 1.5x interquartile range; points, data.

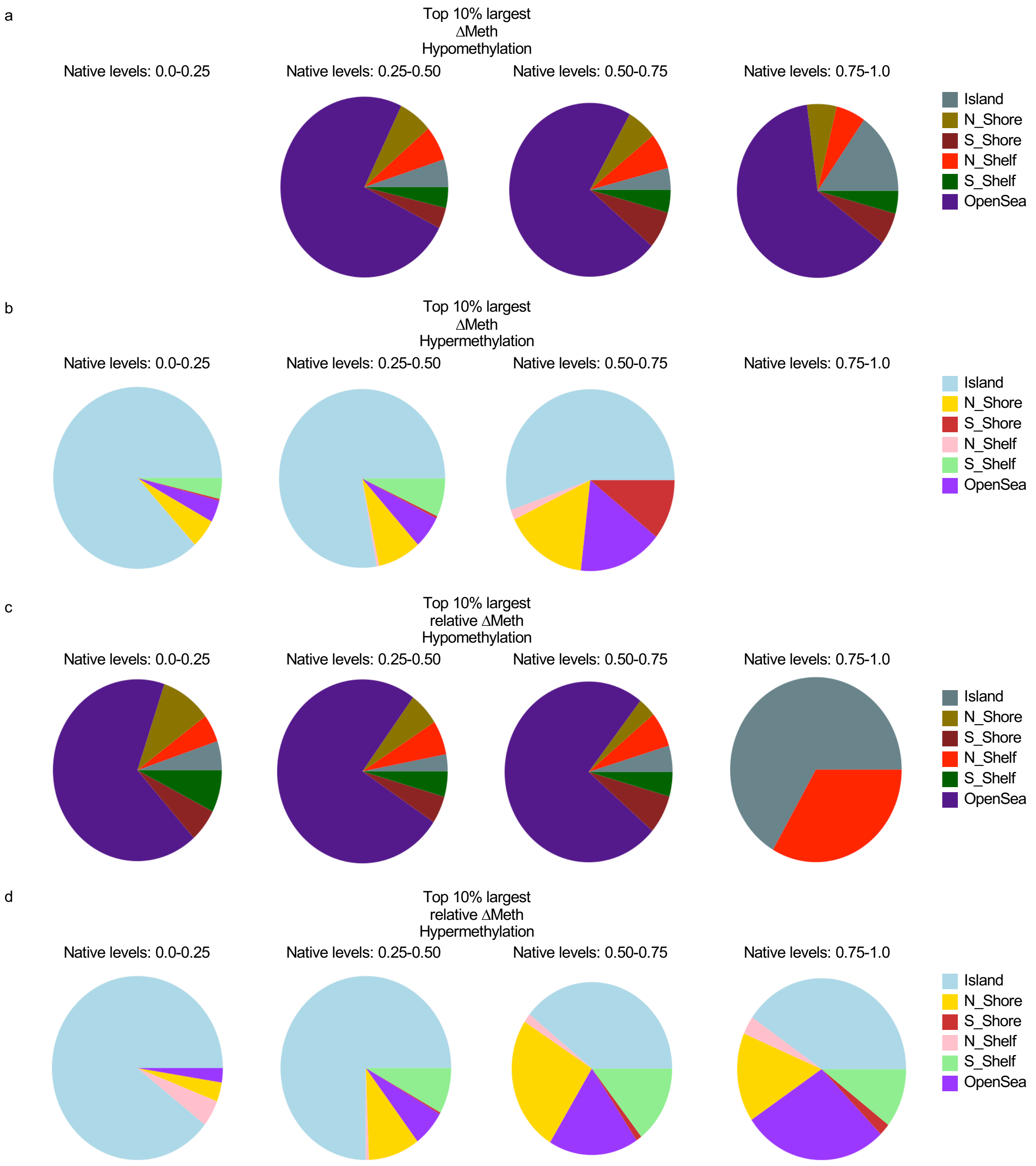

Supplemental Figure 9. The proportions of the different genomic regions in the 10% largest DNA methylation alterations. Pie charts showing the proportion of different genomic regions in top 10% largest ΔMeth in the **a** hypomethylation and **b** hypermethylation directions and the top 10% largest relative ΔMeth in the **c** hypomethylation and **d** hypermethylation directions.

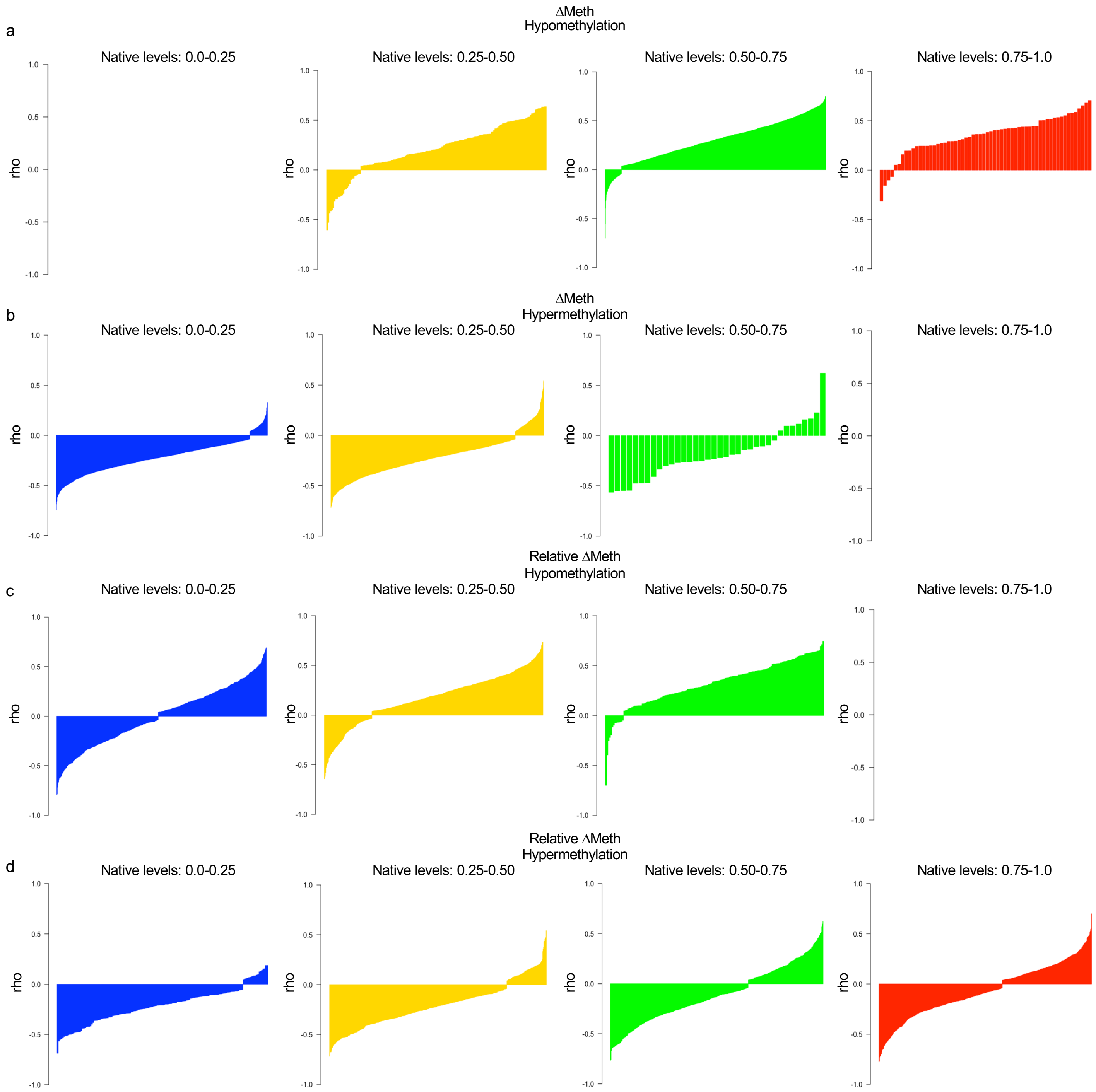

Supplemental Figure 10. Correlation between DNA methylation and gene expression level. Bar plots showing the strength and direction of correlation between DNA methylation and gene expression level identified by using ΔMeth in the **a** hypomethylation and **b** hypermethylation directions and by using relative ΔMeth in the **c** hypomethylation and **d** hypermethylation directions.

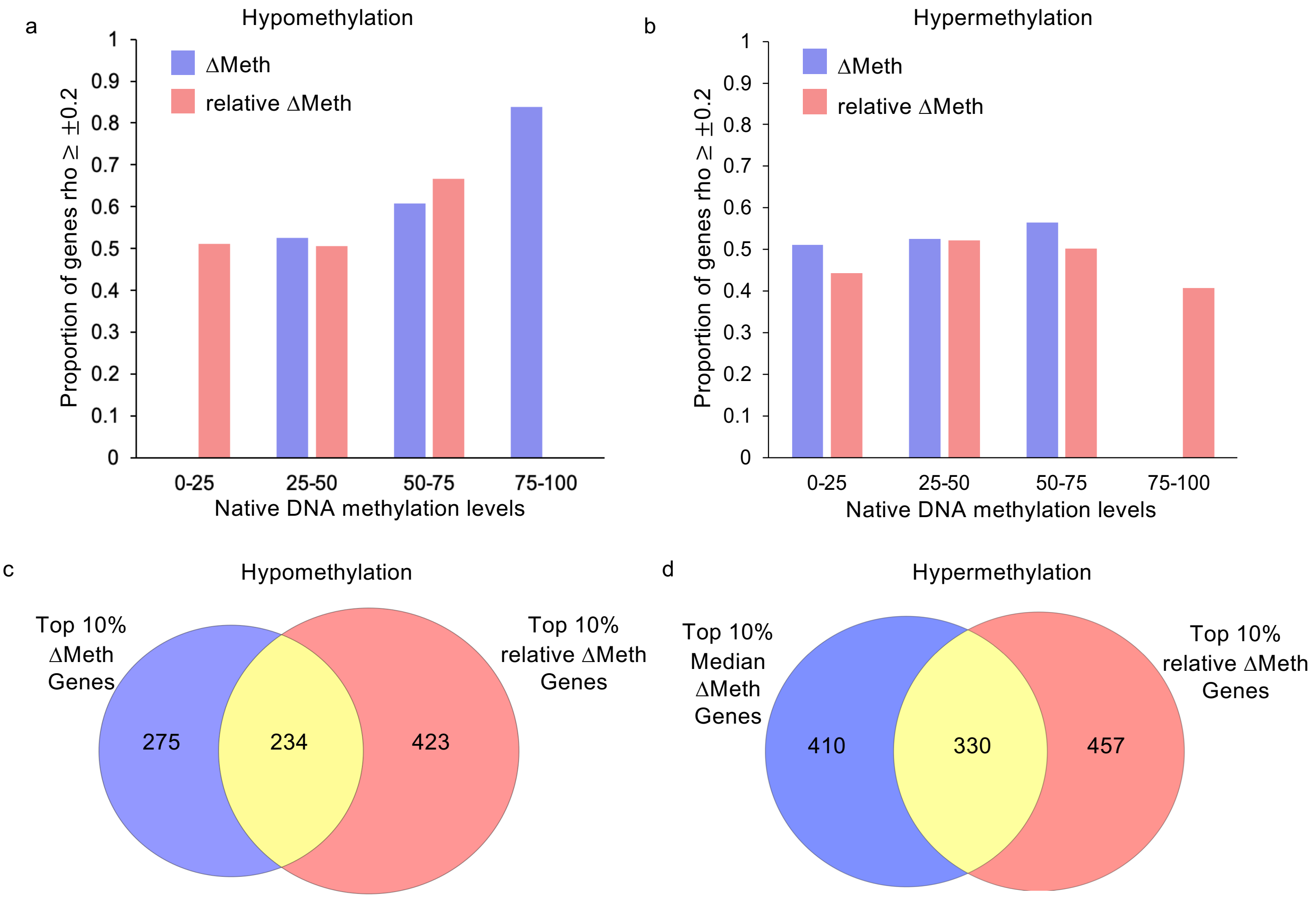

Supplemental Figure 11. Characteristics of the genes that have significant and strong correlation between DNA methylation and RNA expression. Bar plots showing the proportions of genes with strong correlation between RNA expression and DNA methylation levels identified by CpG site with varying levels of native DNA methylation in the **a** hypomethylation and **b** hypermethylation directions. Venn diagrams showing the number of genes that have strong correlation between DNA methylation and RNA expression identified by CpG sites in the **c** hypomethylation and **d** hypermethylation directions.

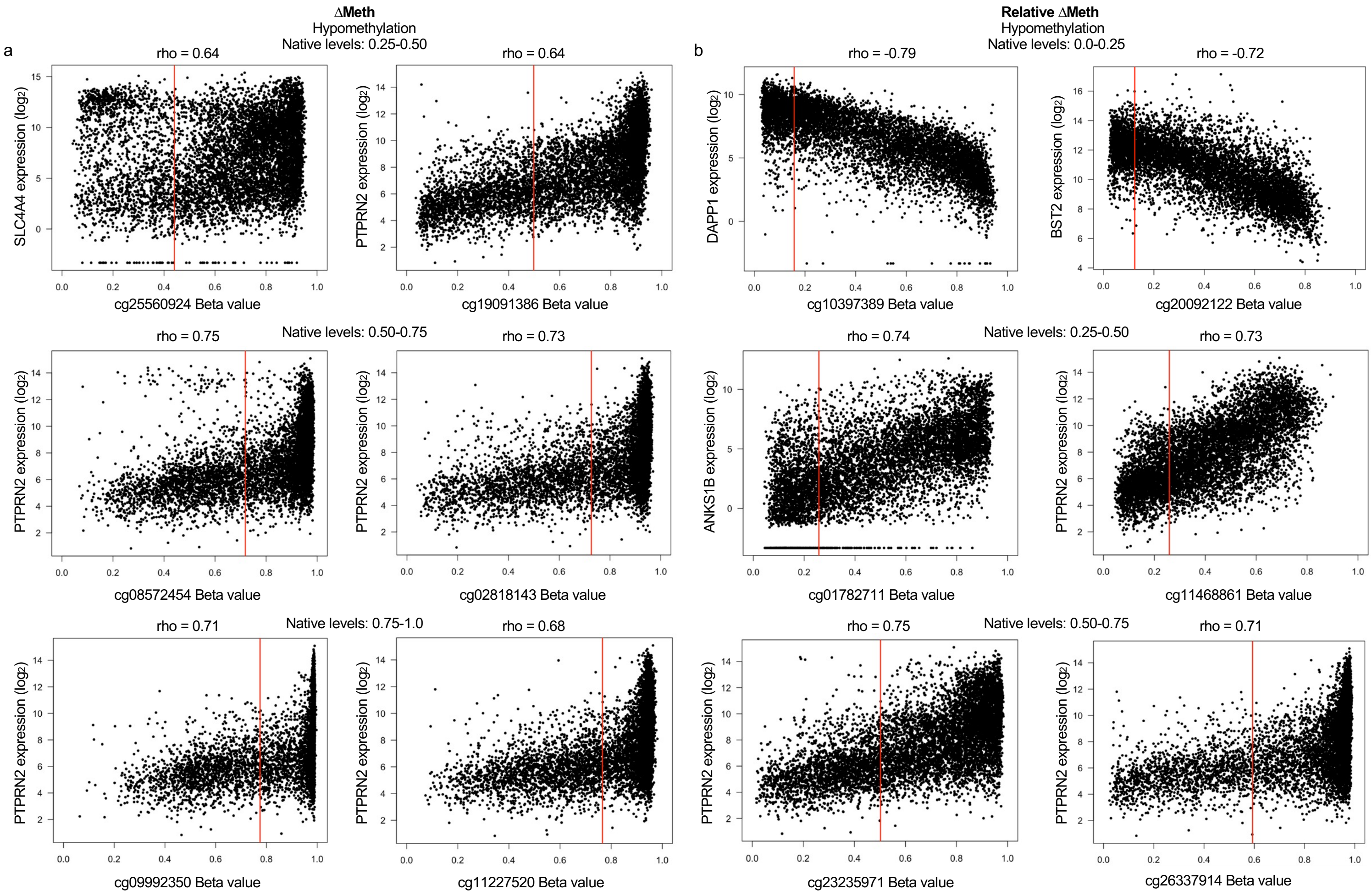

Supplemental Figure 12. Top two hypomethylation alterations with largest correlation from each of the native DNA methylation quantiles. Dot plots showing the DNA methylation and gene expression levels with the top two largest correlations from each of the native DNA methylation quantile intervals identified by using **a** ΔMeth and **b** relative ΔMeth in the hypomethylation direction. Red line indicates the native DNA methylation level. Rho correlation is calculated from the Spearman's correlation.

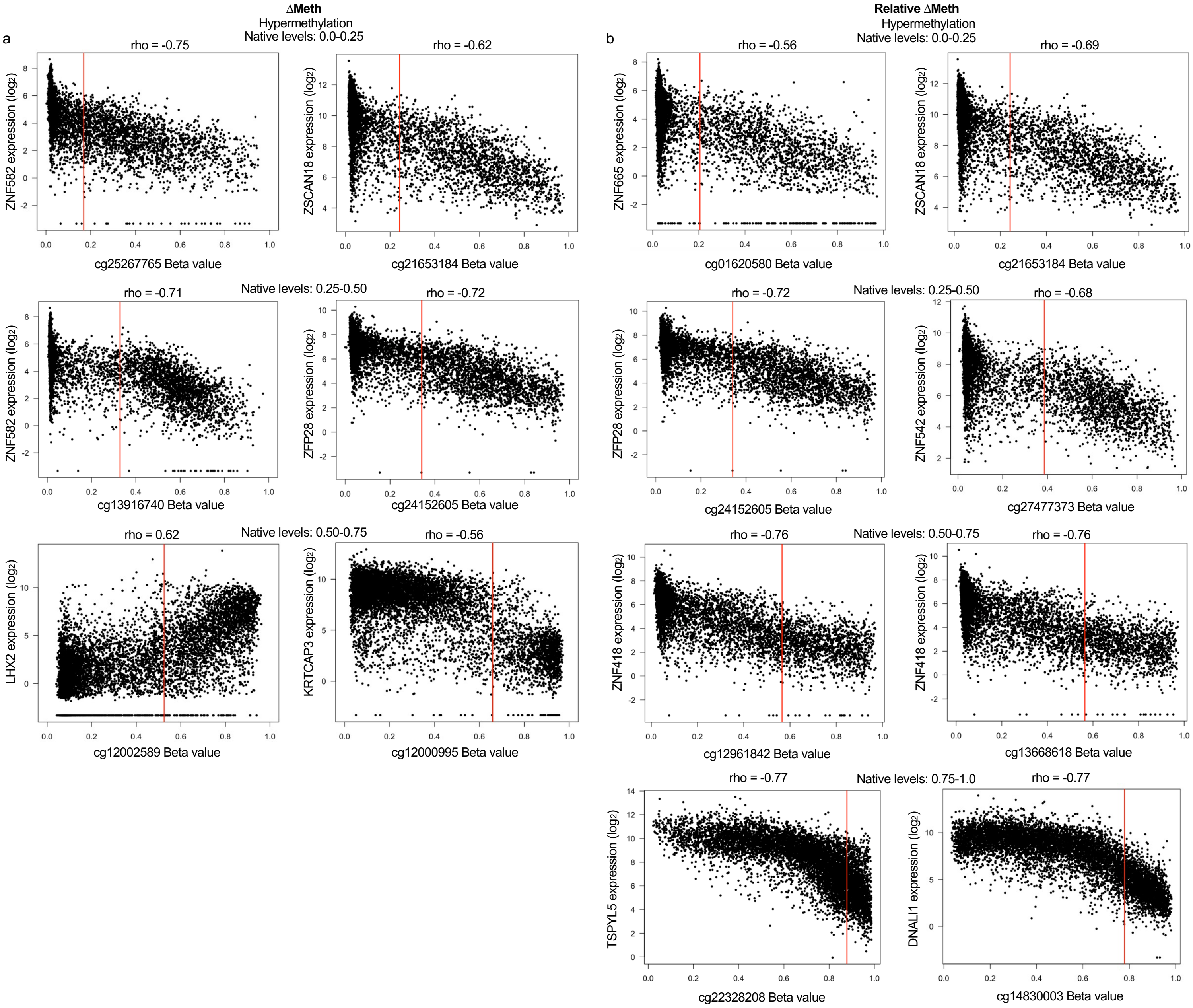

Supplemental Figure 13. Top two hypermethylation alterations with largest correlation from each of the native DNA methylation quantile intervals. Dot plots showing the DNA methylation and gene expression levels with the top two largest correlations from each of the native DNA methylation quantile intervals identified by using **a** ΔMeth and **b** relative ΔMeth in the hypermethylation direction. Red line indicates the native DNA methylation level. Rho correlation is calculated from the Spearman's correlation.

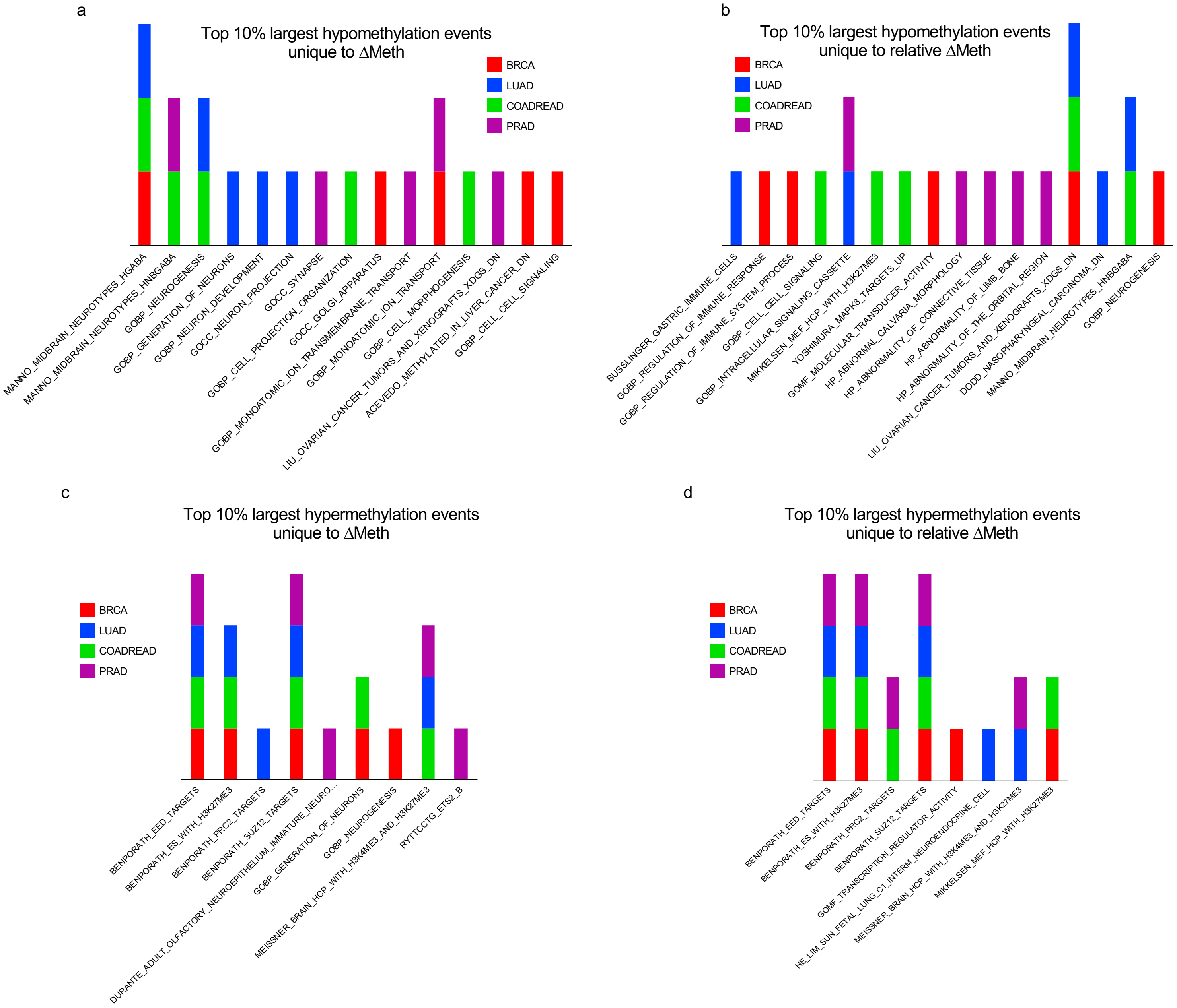

Supplemental Figure 14. GSEA pathway results of individual tumor types. Bar plots showing the top 5 most statistically significant GSEA pathway results (False Discovery Rate (FDR) $P < 0.05$) from the top 10% most frequent and largest alterations in genes with statistically significant correlation between DNA methylation and expression levels uniquely identified in the hypomethylation direction by **a** ΔMeth and **b** relative ΔMeth and in the hypermethylation direction uniquely identified by **c** ΔMeth and **d** relative ΔMeth.

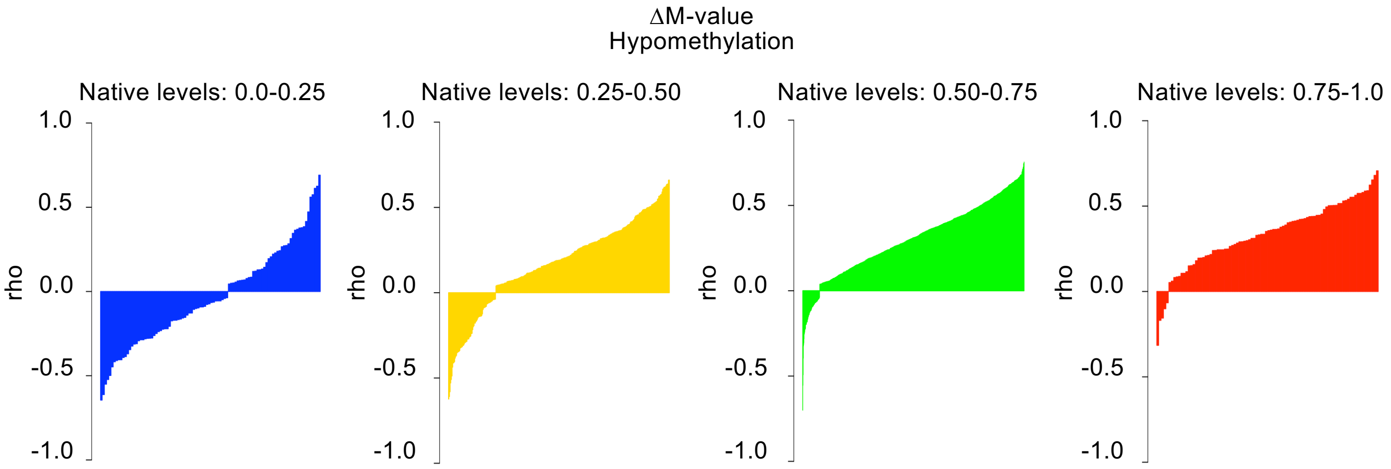

Supplemental Figure 15. Correlation between DNA methylation and gene expression level. Bar plots showing the strength and direction of genes with statistically significant correlation between DNA methylation and gene expression level.
