# Supplementary material for: An unbiased approach to measure aberrant DNA methylation alterations
Source: Nat Commun. 2026 Mar 27;17:4522. doi: 10.1038/s41467-026-71089-5 (PMC13194872; doi:10.1038/s41467-026-71089-5)
Supplement: Supplementary file 4 — Description of Additional Supplementary Files [file 41467_2026_71089_MOESM4_ESM.pdf]

Description of Additional Supplementary Files

Supplementary Data 1

Description: Normal tissue datasets.

Supplementary Data 2

Description: Cancer tissue datasets.

Supplementary Data 3

Description: Top 10% probes identified in the hypomethylation direction.

Supplementary Data 4

Description: Supplemental Table 4. Top 10% probes identified in the hypermethylation direction.

Supplementary Data 5

Description: Genes identified in the hypomethylation direction that have significant correlation between DNA methylation and expression levels.

Supplementary Data 6

Description: Genes identified in the hypermethylation direction that have significant correlation between DNA methylation and expression levels.

Supplementary Data 7

Description: GSEA identified pathways of genes uniquely identified by ∆Meth in the hypomethylation direction.

Supplementary Data 8

Description: GSEA identified pathways of genes uniquely identified by realtive ∆Meth in the hypomethylation direction.

Supplementary Data 9

Description: GSEA identified pathways of genes uniquely identified by both ∆Meth and realtive ∆Meth in the hypomethylation direction.

Supplementary Data 10

Description: GSEA identified pathways of genes uniquely identified by ∆Meth in the hypermethylation direction.

Supplementary Data 11

Description: GSEA identified pathways of genes uniquely identified by realtive ∆Meth in the hypermethylation direction.

Supplementary Data 12

Description: GSEA identified pathways of genes uniquely identified by both ∆Meth and realtive ∆Meth in the hypermethylation direction.

Supplementary Data 13

Description: GSEA identified pathways of genes identified uniquely by ∆M-Value in the hypomethylation direction.

Supplementary Data 14

Description: GSEA identified pathways of genes identified uniquely by relative ∆Meth in the hypomethylation direction.

Supplementary Data 15

Description: GSEA identified pathways of genes identified by both ∆M-Value and relative ∆Meth in the hypomethylation direction.

Supplementary Data 16

Description: GSEA identified pathways of genes uniquely identified by ∆Meth in common HMDs in the hypomethylation direction in breast cancer.

Supplementary Data 17

Description: GSEA identified pathways of genes uniquely identified by relative ΔMeth in common PMDs in the hypomethylation direction in breast cancer.

Supplementary Data 18

Description: GSEA identified pathways of genes uniquely identified by relative ΔMeth in common HMDs in the hypomethylation direction in breast cancer.

Supplementary Data 19

Description: GSEA identified pathways of genes uniquely identified by ΔMeth in common HMDs in the hypomethylation direction in colorectal cancer.

Supplementary Data 20

Description: GSEA identified pathways of genes uniquely identified by relative ΔMeth in neither common PMDs/HMDs in the hypomethylation direction in colorectal cancer.

Supplementary Data 21

Description: GSEA identified pathways of genes uniquely identified by relative ΔMeth in common PMDs in the hypomethylation direction in colorectal cancer.

Supplementary Data 22

Description: GSEA identified pathways of genes uniquely identified by relative ΔMeth in common HMDs in the hypomethylation direction in colorectal cancer.
